# The shell game: how institutional review boards shuffle words

**DOI:** 10.1186/1479-5876-12-201

**Published:** 2014-08-14

**Authors:** Simon N Whitney

**Affiliations:** Department of Family and Community Medicine, Baylor College of Medicine, 3701 Kirby Drive, Suite 600, Houston, TX 77098 USA

**Keywords:** Institutional Review Board, Ethics Committees, Research ethics, Research, Government Regulation, Informed consent, Biomedical research/ethics, Ethical review

## Abstract

Concepts like coercion, vulnerability, and dignitary harm have acquired specialized meanings in the research ethics literature. Institutional Review Boards (IRBs), also called Research Ethics Committees (RECs), sometimes use these concepts in two different ways without acknowledging or even realizing what they are doing. IRBs mislabel any language that encourages subject participation in trials as “coercive,” then demand its removal as if it were actually coercive in the sense of a threat of force. An example of language that is treated as coercive is the use of the word “hope” in an educational brochure about clinical trials. The concepts of vulnerability and dignitary harm are similarly misused. The regulations instruct IRBs to protect vulnerable groups; but IRBs sometimes use a group’s vulnerability to one threat to protect it against an unrelated and harmless threat, as when homeless people, who are vulnerable to street crime and disease, are protected from the risk of an interview. Finally, the term “dignitary harm” is so vague that IRBs can use it to restrict research that is entirely free of risk, while ignoring the possibility that research might provide the dignitary benefit of contributing to society’s health and welfare. Dignitary harm—usually nonphysical “harm” of which the subject is entirely unaware—can be deemed more important than obtaining information that subjects want or actual risk of physical injury. These vague or shifting definitions permit the IRB to play a shell game without either the board or the investigator realizing what is happening.

## Background

Responsible regulation of research by Institutional Review Boards (IRBs) or Research Ethics Committees (RECs) requires a shared understanding, between regulator and scientist, of what critical terms mean; it stumbles when key concepts, like coercion, vulnerability, and dignitary harm, have vague or shifting definitions. This is one piece of the much larger problem of dysfunctional regulatory review, documented in recent books [1, 2] and hundreds of articles.

## Coercion

One concept that IRBs routinely misuse is coercion; the experience of Kenneth Getz is a recent case in point. Getz, who directs the Center for Information and Study on Clinical Research Participation, wrote a brochure explaining the typical risks and benefits of clinical trial participation. His goal was educational and the brochure did not promote, or even mention, any specific study. Yet an IRB objected to his discussion of how subjects hope to benefit from trial participation and told him that the word “hope” was coercive. The result was a frustrated educator who inveighed against “IRB bureaucracy and despotism” [3] and, no doubt, an IRB that felt the educator does not understand ethical thought.

The Belmont Report, one of the foundational documents of research regulation, restricts “coercion” to the use of “an overt threat of harm [4]”. This is consistent with *Webster*’s, which specifies the use of “force or intimidation” and the *Law Dictionary*’s “compulsion; force; duress [5, 6]”. The distinguished ethicists Ruth Faden and Thomas Beauchamp favor a slightly broader approach, proposing that, in the context of informed consent, coercion requires “a credible threat of unwanted and unavoidable harm so severe that the person is unable to resist acting to avoid it [7]”. There is much more to be said, of course; Jonathan Baron, Alan Wertheimer, and Barbara Evans have presented penetrating analyses of the topic [8–10].

Popular IRB guides ignore these subtleties and mangle the standard definitions. One handbook claims that “coercion means that a person is to some degree forced, or at least strongly pushed, to do something that is not good for him or her to do. In discussions of research regulation the term ‘undue influence’ is often used to describe the concept of coercion [11]”. This manual thus expands the narrow concept of coercion to include persuasion.

A second handbook agrees: “Coercion can be subtle: persuasion, argument, and personality can be used to compel an individual to act in a certain way…. Coercion—including all the subtle forms—has no place in research [12]”. There is, of course, no such thing as subtle coercion. A guide to IRB management and function claims that in recruitment for clinical trials, “the possibilities for misinforming or disinforming potential subjects abound” and “the possibilities for inadvertent, unintentional coercion, or undue influence are also high [13]”. Inadvertent or unintentional coercion is oxymoronic.

With encouragement from these guides, IRBs reject the standard meaning of the word and use “coercion” to refer to any statement, however innocuous, that might encourage trial participation. Some IRBs believe, for instance, that it is coercive for a consent form to mention that a study is funded by the National Institutes of Health.

One example of the misuse of the concept of coercion is in the acronyms of clinical trials. Investigators have long realized that it is better to refer to a study as, for instance, CAST instead of the Cardiac Arrhythmia Suppression Trial; there are now thousands of acronymic trials. The research ethics literature once ignored these acronyms, then began to criticize them as potentially coercive, and now presents that hypothesis as established fact.

James Orlowski and James Christensen may have been the first to argue that the acronyms of protocols, like HELP or HOPE, might be coercive in themselves, writing in 2002 that acronyms “may be subtly playing on the hopes or dreams of research subjects, a form of coercion [14]”. By 2013, the “potentially coercive” nature of acronyms had been transformed, without proof, from speculation to fact in the *Textbook of Pharmaceutical Medicine*, which states flatly that study acronyms like CURE, HOPE and HELP “can entice a subject to give consent [15]”. Optimism and coercion are thus united.

IRBs are alert for coercion in the protocols they review, and, when coercion is defined so liberally, they readily find it. Without realizing it, IRBs then substitute the narrow meaning of the word for the broad one. Coercion by force or threat of force would of course be unacceptable; this substitution of meanings gives IRBs carte blanche to interfere with a wide range of investigator actions they deem coercive. This is why Getz was forbidden to mention hope in an educational brochure describing clinical trials, a decision he found ridiculous. But his opinion was irrelevant, since from the IRB determination there was no appeal.

## Vulnerability

The regulations instruct IRBs to provide “additional safeguards” when “some or all of the subjects are likely to be vulnerable to coercion or undue influence, such as children, prisoners, pregnant women, mentally disabled persons, or economically or educationally disadvantaged persons ….” [16] Vulnerable groups are said to need additional protections against coercion, however loosely both terms are used. Vulnerability itself is only casually defined and can be limitlessly expanded, for who is not subject to influence?

The shell game here is in the shifting meaning of the label “vulnerable.” A patient with AIDS is vulnerable to tuberculosis; a prisoner is vulnerable to the warden. These are different kinds of vulnerability. The homeless are vulnerable to violence on the street but not to advertisements that seductively suggest the glamour of a Rolex on your wrist. IRBs go astray when they determine that a group of subjects is vulnerable to one harm and, based on that, pivot and protect that group against an unrelated hazard that poses no threat to their welfare.

So, for instance, one IRB’s community representative resisted a proposal to study the homeless “because she felt that the population was too easily exploited.” The homeless might be vulnerable to offers of shelter and food in exchange for participation in risky research; but this scientist wanted merely to conduct interviews, a harmless process that might aid the group [17].

When an IRB demands changes or rejects a proposal because it considers the subjects to be vulnerable, the investigator may disagree, believing that any vulnerability is irrelevant to the proposed research; but no matter. The IRB reserves the right to make its own, definitive, judgment.

## Dignitary harm

Dignitary harm is not mentioned in the regulations governing IRBs; it came to prominence with the 2001 report of the National Bioethics Advisory Commission (NBAC). Dignitary harms, announces NBAC, occur “when individuals are not treated as persons with their own values, preferences, and commitments, but rather as mere means, not deserving of respect [18]”.

The nebulous concept of dignitary harm can lead to pernicious regulatory guidance. Consider, for example, a subject who is enrolled in a randomized controlled trial without providing a fully informed consent, as might happen in a study of an emergency condition like cardiac arrest or a stroke. As Norman Fost notes, in the absence of known effective therapy, innovative care under the direction of the subject’s own doctor (“essentially unreviewed, uncontrolled experimentation”) would be an inferior alternative to participation in the formal trial [19].

Morris and Nelson, discussing these options, concede that although innovative care is “perhaps less safe” than a controlled trial, enrolling subjects in the trial without full informed consent may represent a dignitary harm. The reason: if these subjects were fully informed, they might be upset at being randomized, or at being assigned to a study arm later proven to be inferior, or might believe that study participation was more harmful (although it is not), or might even be dismayed to learn of “the inadequacy of current medical knowledge [20]”. Enrollment in a controlled trial that is safer is thus cast in an ominous moral shadow because of irrational concerns that the subject might or might not have.

Individual IRBs are free to follow analogous reasoning as they review individual protocols, invoking the malleable concept of dignitary harm to justify intervention to protect subjects from hypothetical or speculative risks. Dignitary harm can be invoked whenever something seems wrong to the board but no actual harm has occurred or been threatened. Consider, for instance, large-scale database research which cannot be done if consent is required. NBAC considers research without consent to constitute a dignitary harm—one that the subject never knows occurred [18]. This “harm” may cloak a practical benefit, as when scientists, by examining thousands of records, can determine the subjects’ risk of cancer, but dignitary harm may trump actual benefit [21].

Dignitary harm is an infinitely flexible concept, and because it has no fixed meaning, an IRB can define it any way it likes and the researcher cannot rebut it. We might, instead, consider the possibility that participating in research intended to improve the lives of one’s fellows should be considered a dignitary benefit. But risk dominates benefit in this regulatory system.

## Conclusion

### The baffled scientist

These variations on the shell game are a prime cause of anger among investigators, who feel badly treated—like any other shell game victim—but are unable to put their finger on what went wrong.

I am not accusing IRBs of hypocrisy. They appear sincere in believing that these concepts are used in a special sense in the context of research regulation, and that investigators simply do not understand how research ethics works. But malice is irrelevant. The shell game is destructive whether the operator is a shark or a suit.

## Author’s information

SW holds the William O’Donnell and Regina O’Donnell Chair in Family Medicine in the Department of Family and Community Medicine at Baylor College of Medicine. He served on the Stanford IRB in 1997–1998.
